# Acquired and Inherited Zinc Deficiency-Related Diseases in Children: A Case Series and a Narrative Review

**DOI:** 10.3390/pediatric16030051

**Published:** 2024-07-25

**Authors:** Tommaso Bellini, Marta Bustaffa, Barbara Tubino, Benedetta Giordano, Clelia Formigoni, Elena Fueri, Federica Casabona, Barbara Vanorio, Andrea Pastorino, Astrid Herzum, Caterina Matucci-Cerinic, Serena Arrigo, Gianmaria Viglizzo, Emanuela Piccotti

**Affiliations:** 1 Pediatric Emergency Room and Emergency Medicine Unit, Emergency Department, IRCCS Istituto Giannina Gaslini, 16147 Genoa, Italy; martabustaffa@gaslini.org (M.B.); barbaratubino@gaslini.org (B.T.); emanuelapiccotti@gaslini.org (E.P.); 2 Department of Neuroscience, Rehabilitation, Ophthalmology, Genetics, Maternal, and Child Health (DINOGMI), University of Genoa, 16132 Genoa, Italy; 4043931@studenti.unige.it (B.G.); 3805091@studenti.unige.it (C.F.); 3764730@studenti.unige.it (E.F.); 3907391@studenti.unige.it (F.C.); 5749021@studenti.unige.it (B.V.); 5288131@studenti.unige.it (A.P.); 3 Dermatology Unit, IRCCS Istituto Giannina Gaslini, 16147 Genoa, Italy; astridherzum@gaslini.org (A.H.); gianmariaviglizzo@gaslini.org (G.V.); 4 Reumatology and Autoinflammatory Diseases Unit, IRCCS Istituto Giannina Gaslini, 16147 Genoa, Italy; caterinamatuccicerinic@gaslini.org; 5 Gastroenterology and Digestive Endoscopy Unit, IRCCS Istituto Giannina Gaslini, 16147 Genoa, Italy; serenaarrigo@gaslini.org

**Keywords:** acrodermatitis enteropathica, transient neonatal zinc deficiency, acquired zinc deficiency, metabolic diseases, dermatitis, pediatric emergency departments

## Abstract

Zinc deficiency is a significant global health concern among children, manifesting in various acquired and inherited conditions. This comprehensive overview of acquired and inherited zinc deficiency-related diseases in children aimed to explore the clinical presentations, diagnostic challenges, and management strategies associated with these conditions. This case series elucidates the diverse clinical manifestations of zinc deficiency in pediatric patients, ranging from dermatitis and growth retardation to immune dysregulation and neurological abnormalities, and discusses the underlying genetic mechanisms, clinical phenotypes, and therapeutic interventions. The complexity of zinc deficiency-related diseases in children underscores the need for a multidisciplinary approach involving pediatricians, dermatologists, geneticists, and nutritionists to optimize patient care and outcomes.

## 1. Introduction

Zinc is an essential trace element involved in numerous physiological processes, including cellular proliferation, wound healing, and DNA synthesis, serving as a cofactor for over 1000 enzymatic reactions and modulating gene expression, immune function, and oxidative stress response [1,2,3,4,5,6,7,8]. It also plays a key role in the correct functioning and development of the immune, nervous, and endocrine systems during fetal life, childhood, and adolescence [2,3,4,5,7,8,9].

Zinc is the second most abundant trace mineral in the human body after iron, and its deficiency is a significant public health concern, particularly in pediatric populations owing to its pivotal role in growth and development. Nearly 20% of the global population is affected by mild-to-moderate zinc deficiency, particularly in low-income countries, where diets lacking animal-derived foods and zinc-rich sources contribute to zinc insufficiency [1,2,3,4,8,9,10,11,12]. It has been estimated that in some low- and middle-income countries, almost half of all children might have zinc deficiency [12].

Zinc deficiency in pediatric patients may arise from various etiological factors, including inadequate dietary intake and/or increased demand, malabsorption syndromes, excessive losses, chronic illnesses, and genetic disorders affecting zinc metabolism [2]. Table 1 shows the main diseases associated with these etiological mechanisms.

The aim of this manuscript is to present a schematic review of the pathophysiology of zinc deficiency, its associated diseases, clinical features, differential diagnoses, and management.

## 2. Case Series

### 2.1. Case One

**Figure 1:**
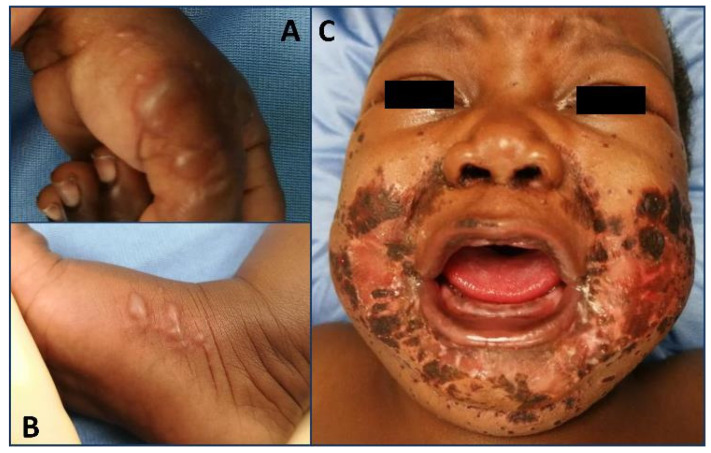
(**A**) Bullous lesions of the fingers with spared nails. (**B**) Acral bullous lesions in the feet. (**C**) Erosive, partially crusted erythematous plaques, sharply demarcated and symmetrically distributed impetiginized lesions on the cheeks, ears, neck, and chin.

A previously full-term healthy three-month-old female presented to the pediatric emergency department (PED) with a three-week history of progressively worsening skin lesions and was unresponsive to topical antibiotic and antifungal treatment. Moreover, she experienced fever, irritability, and a lack of appetite four days prior to PED admission, but she did not present with diarrhea. She was exclusively breastfed, and her past medical history was unremarkable. The parents of the patient were non-consanguineous. The mother revealed that the eldest son presented with similar skin lesions during breastfeeding. The patient was irritable and had symmetric, sharply demarcated, erythematous, erosive impetiginized plaques with scaling and crusting on the cheeks, auricles, neck, toes, and genital area, with bilateral interdigital bullous lesions (Figure 1). There was no evidence of alopecia, and the nails were spared. *Pseudomonas Aeruginosa* skin colonization was detected; thus, she was hospitalized and started broad-spectrum intravenous antibiotic therapy. The diagnosis of acrodermatitis enteropathica (AE) was considered and further supported by low plasma zinc levels in two samples (21 and 32 µg/mL; reference range, 68–107 µg/mL) and alkaline phosphatase (42 U/L; reference range, 122–469 U/L). Zinc supplementation was initiated at a dose of 0.5 mg/kg/day. The skin lesions resolved within two weeks, and the zinc levels returned to normal. However, the *SLC39A4* gene analysis was negative. Due to the response to low-dose zinc therapy, the negativity of genetic analysis, and a family history of similar symptoms in the older brother, transient neonatal zinc deficiency (TNZD) was suspected. However, the mother refused further genetic investigation and the patient was lost to follow-up.

### 2.2. Case Two

**Figure 2:**
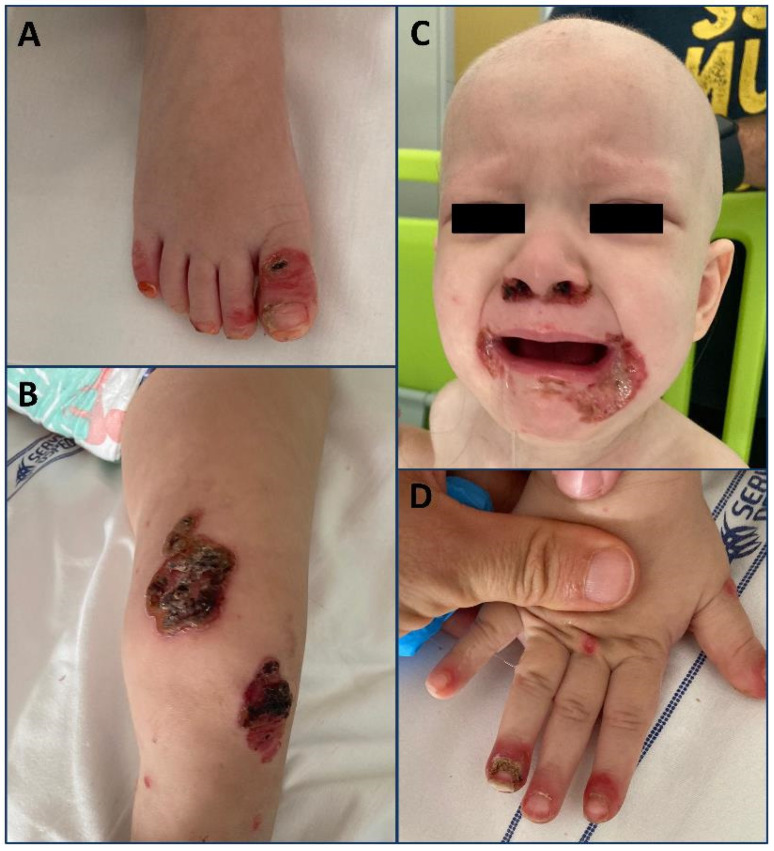
(**A**) Crusty and hyperemic rash on the feet, with paronychia. (**B**) Erosive, crusted, and bullous perioral lesions with alopecia universalis. (**C**) Acral crusted lesions affecting the knees. (**D**) Paronychia and onychodystrophy affecting the fingers.

An 18-month-old girl born to non-consanguineous parents presented to the PED with a 4-month history of crusted, erosive, and bullous lesions with a perioral, periocular, and acral distribution (Figure 2). Universal alopecia was also observed. She was exclusively breastfed until the age of 6 months, and the remaining remote medical history was unremarkable. Furthermore, no diarrhea was reported. She had been previously evaluated at another hospital, and due to a skin swab positive for *Staphylococcus Aureus*, she had been treated with topical antibiotics and antifungals without any improvement. When epidermolysis bullosa was suspected, a skin biopsy was performed, revealing an aspecific vacuolar degeneration of keratinocytes with dermal edema and lymphocytic infiltration. Thus, zinc deficiency was suspected, confirmed by low zincemia in two determinations (44 and 30 µg/mL; reference range, 68–107 µg/mL), along with low alkaline phosphatase (68 U/L; reference range, 122–469 U/L). Secondary causes of zinc deficiency, such as intestinal malabsorption and celiac disease, were excluded; therefore, genetic testing for acrodermatitis enteropathica was performed and is still ongoing. Zinc supplementation therapy (3 mg/kg/day) was initiated with an improvement in skin lesions, zinc levels, and alkaline phosphatase values. After three months of therapy, the lesions had completely healed, and the alopecia had resolved.

### 2.3. Case Three

**Figure 3:**
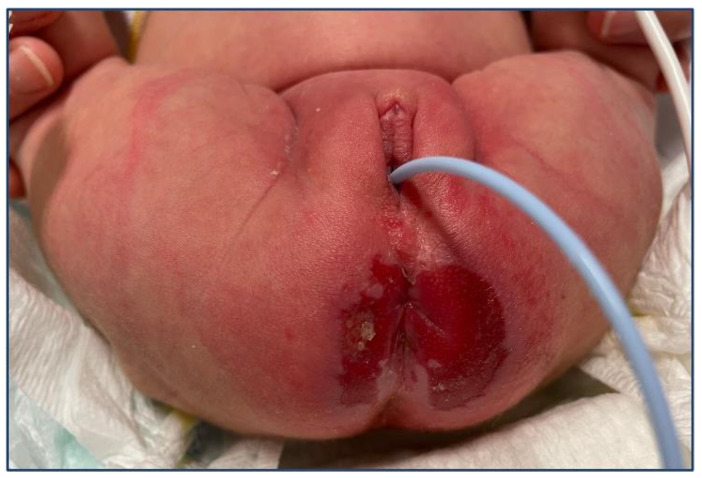
Erythematous erosive perineal plaque.

A 20-day-old girl was admitted to the PED because of a demarcated erythematous erosive perineal plaque (Figure 3). She was born late preterm at 35 weeks + 6 days and was small for gestational age (<3° percentile). The patient was exclusively breastfed, but her weight remained stable for 20 days. Owing to a previous positive skin swab test for *Enterobacter cloacae*, local topical antibiotic therapy was initiated with no clinical improvement. Zinc levels were reduced (55 µg/mL; reference range, 68–107 µg/mL), and other metabolic causes of growth deficiency were excluded. Zinc therapy (1.5 mg/kg/d) was initiated along with artificial milk supplementation, with resolution of skin lesions and resumption of weight gain. Genetic tests for acrodermatitis enteropathica were not performed.

### 2.4. Case Four

A 14-month-old male child presented to the emergency room with perioral and limb vesicular skin lesions since the age of seven months. Previously healthy, he was exclusively breastfed until he was six months old and then weaned. The patient did not present any other symptoms. A skin biopsy was performed with evidence of epidermal psoriasiform hyperplasia associated with superficial perivascular dermatitis. Under the suspicion of acrodermatitis enteropathica, zincemia was measured and reduced in two determinations (18 and 30 µg/dL, respectively; reference range, 68–107 µg/mL). Alkaline phosphatase levels were also reduced (61 and 80 U/L; reference range 142–335). To confirm the diagnosis, genetic testing of *SLC39A4* was performed, highlighting the pathogenic variant c.295G > Ap in homozygosis. Supplementary therapy with zinc at a dose of 3 mg/kg/day was then administered with complete resolution of the skin findings. The parents denied taking photos of the skin lesions.

## 3. Materials and Methods

A systematic literature search was conducted using the *Pubmed* database to identify relevant articles. Four researchers independently conducted the search process to ensure comprehensive coverage and included keywords related to acrodermatitis enterophatica and zinc deficiency in pediatric patients. Articles published in the last 10 years were included in the search. Search terms were selected based on their relevance to the research question and combined using Boolean operators. Filters were used to limit the search to articles written in English and published in peer-reviewed journals. Additionally, manual searches were performed by screening the reference lists of relevant articles. Duplicates were identified and removed using reference management software, and any discrepancies in article selection were resolved through consensus. The final selection of articles was based on their relevance to the research question and the quality of evidence presented. Data extraction was performed independently by four reviewers and crosschecked for accuracy.

## 4. Zinc Homeostasis

Zinc is found in many dairy products such as crustaceans (oysters, crabs), meat (beef, turkey, chicken, pork), tree nuts (cashews, almonds), legumes (beans), peanuts, and whole grains [13].

The human body contains a total of 2–3 g of zinc [1]. It is absorbed in the small intestine through a specific zinc transporter called ZIP4 and then released into the bloodstream by another zinc transporter family called ZnT [1,2,8]. Once absorbed, all zinc is bound to albumin for 80% and alpha2-macroglobulin for 20% and is transported to the liver and stored in muscles and bones (80–85%) and in the skin and liver (10%) [1,2,7,8]. Thus, plasma zinc may not reflect the total body zinc status, representing only 0.1% of the body’s total zinc stores [8,13].

Zinc homeostasis is primarily dependent on the interplay between intestinal zinc absorption and excretion of endogenous intestinal zinc, with marginal involvement of the kidneys and bones [8]. The absorption of dietary zinc is best characterized as a saturable process that occurs in the small intestine, primarily determined by the amount of zinc ingested, and may be independent of the zinc status of the host [4,7,8].

## 5. Etiology

Zinc deficiency can be inherited or acquired. All of the conditions described below disrupt zinc homeostasis, impair zinc utilization, or disrupt metabolic pathways crucial for skin integrity and are briefly described from here onwards.

### 5.1. Primary Zinc Deficiency

The most likely scenario for pure dietary zinc deficiency is the older breastfed infant, especially those who have been exclusively breastfed [4]. The zinc content of human milk starts at high concentrations (>3 mg/L) and progressively declines postpartum to <1 mg/L within six months [4,5]. Interestingly, although the underlying biological mechanisms are not yet fully understood, zinc concentration in breast milk is independent of maternal dietary intake [4,7]. For these reasons, the six-month-old breastfed infant becomes dependent on other sources of zinc, typically complementary foods and meat [4].

Although the concentration of zinc in bovine milk is similar to that in human milk, in bovine milk there is another ligand with a higher molecular weight that cannot improve zinc availability in the intestinal lumen, thus impairing its absorption. This explains why AE manifestations begin after weaning and switch to bovine milk [14].

### 5.2. Genetically Based Zinc Deficiency

#### 5.2.1. Acrodermatis Enteropathica

Genetic disorders affecting zinc transporters or intracellular zinc utilization pathways can also lead to inherited zinc deficiency despite adequate dietary intake. AE is a rare autosomal recessive disorder, with an incidence rate of 1:500,000 and no apparent predilection for race or sex [3,15,16]. The consanguinity of parents may represent a precious anamnestic clue [4,17]. In most cases, AE is caused by mutations in *SLC39A4*, located on chromosome 8q24.3, which encodes the zinc transporter protein ZIP4 [2,4,15]. ZIP4 plays a crucial role in the intestinal absorption of zinc, and its loss of function impairs zinc uptake, leading to systemic zinc deficiency and characteristic clinical features of AE [3,18]. Patients with the most severe AEs are homozygous for a frameshift mutation that induces a premature termination codon lacking the last five transmembrane domains, although there is still no consensus on the genotype–phenotype correlations [4,12,19]. In other rare instances, AE may result from mutations in *SLC39A13*, which encodes another zinc transporter protein, ZIP13 [2].

#### 5.2.2. Transient Zinc Deficiency

Transient neonatal zinc deficiency (TNZD) is caused by autosomal dominant mutations in SLC30A2, leading to defective zinc transfer from the mammary epithelium to breastmilk [4,5,6,20,21]. This mutation leads to markedly low milk concentrations (<25% of normal values). The exact prevalence of TNZD is unknown, but it may be underdiagnosed, and reports are especially common for infants being delivered prematurely or with low birth weight because of their negative zinc balance (see below) [4,22]. However, Case 1 shows that TNZD may also occur in full-term infants.

### 5.3. Acquired Zinc Deficiency

#### 5.3.1. Premature Infants

Zinc deficiency is more common in premature infants for several reasons that may be combined [4,7]. First, most mother–fetus zinc transfer occurs in the last ten weeks of gestation; thus, premature delivery could reduce zinc storage [2]. Second, premature infants may have a negative zinc balance until the 60th day of life, probably because their immature gut and kidneys have a reduced capacity to absorb zinc [2]. Third, preterm newborns require higher zinc levels because of their rapid growth and development [4,5,22].

Finally, the pattern of declining zinc concentrations in the early postpartum months does not differ between preterm and term human milk; thus, as a premature infant approaches 40 weeks post-conception, the decline in milk zinc concentrations will already have been substantial [2,4].

Signs of deficiency include growth impairment (linear and ponderal) and characteristic dermatitis similar to that described for AE and, because of the vulnerability and complexity of this patient group, zinc deficiency should be considered in any preterm infant who is not growing well, despite apparently adequate calories and macronutrient intake [4].

Patients born at term but with intrauterine growth retardation and/or low birth weight also represent a category of patients at risk of zinc deficiency due to inadequate storage during pregnancy [1,6,19,22,23,24].

Thus, for all preterm infants predominantly breastfed after discharge, zinc supplementation should be considered [4].

#### 5.3.2. Malabsorption Syndromes

Any disease that can affect intestinal function may cause zinc deficiency [4,8].

Acute diarrhea

Infectious diarrhea can cause zinc malabsorption, especially in developing countries. For this reason, the World Health Organization suggests the administration of zinc for acute persistent diarrhea, especially among the world’s most vulnerable children [1,4,8,25].

Celiac disease

This relatively common condition is an immune-mediated systemic disorder elicited by dietary gluten in genetically susceptible individuals with variable degrees of small intestinal mucosal erosions. The clinical presentation of celiac disease, especially in young infants, may overlap with clinical features of zinc deficiency, such as anorexia, diarrhea, and failure to thrive [4]. It has been reported that patients with celiac disease have both impaired zinc absorption and increased zinc fecal losses [4]. Following a gluten-free diet, all patients should present normalization of plasma zinc levels, even without zinc supplementation [3,4].

Cystic fibrosis

The gastrointestinal manifestations of this common heritable condition are secondary to exocrine pancreatic insufficiency and intestinal mucosal abnormalities, leading to malabsorption and steatorrhea, which may increase the onset of cystic fibrosis in up to 10% of patients [26]. In settings without newborn screening, the presentation of cystic fibrosis may be late and subtle, with associated growth faltering despite adequate calorie intake, diarrhea, and dermatitis similar to AE [3,4,26].

Liver diseases

More than 80% of zinc is carried by albumin; therefore, any liver disease, acute or chronic, which causes protein underproduction can lead to zinc deficiency [3,12,27].

Exocrine pancreatic insufficiency

Zinc is carried predominantly by albumin and alpha-2-macroglobulin, and poor protein absorption due to pancreatic proteolytic enzyme deficiency, regardless of its cause, can lead to zinc deficiency [12,28,29].

Other intestinal diseases

It has been described that other pathologies affecting the intestinal tract, such as inflammatory bowel disease and hypereosinophilic syndrome with eosinophilic gastroenteritis, can reduce the absorption of zinc in the small intestine [28,30,31].

Iatrogenic causes

Prolonged use of chelating agents and medications, such as phytates, ethylenediaminetetraacetic acid, penicillamine, diuretics, and sodium valproate, can interact with zinc in the small intestine, decreasing its absorption [1,2,8,12].

Surgery

Although rare in pediatric patients, pancreaticoduodenectomy can induce zinc deficiency, compromising its absorption in the duodenum and proximal jejunum and leading to pancreatic proteolytic enzyme deficiency [29]. Sleeve gastrectomy, Roux-en-Y gastric bypass, and biliopancreatic diversion performed in bariatric surgery may also cause zinc deficiency due to malabsorption [28,32].

#### 5.3.3. Parenteral Nutrition

Patients on total parenteral nutrition (TPN) are at a unique risk of developing iatrogenic zinc and other nutritional trace element deficiencies, which may be under-recognized [11,27,33]. The zinc deficiency associated with parenteral nutrition may be multifactorial. The primary cause could be inadequate parenteral supplementation of zinc; however, parenteral nutrition often contains cysteine supplementation to maximize parenteral calcium and phosphate provisions that may raise urinary zinc losses by increasing proximal tubular zinc secretion [5,23]. Finally, patients may be dependent on TPN owing to short bowel syndrome, presenting two pathogenetic mechanisms: parenteral deficiency and malabsorption [11,34].

#### 5.3.4. Malnutrition

Acquired AE may present at any age and has been associated with many causes of malnutrition, including veganism, vegetarianism, food faddism, alcoholism, and anorexia nervosa [28,31,35]. A ketogenic diet, which can be offered to patients with drug-resistant epilepsy, is also abused by those who want to take advantage of its proven ability to cause rapid and substantial weight loss [7,28,36]. Children with hypercatabolism have high zinc requirements owing to their elevated zinc losses [33].

#### 5.3.5. Other Zinc Losses

Although rare, some patients may present with increased renal loss of zinc [5]. Nephrotic syndrome and other renal diseases cause osmotic diuresis of zinc via a mechanism similar to that of diabetes mellitus. Furthermore, it causes hypoalbuminemia with a greater free quota of freely filtered zinc [5,10,21,24]. In adult patients, drug abuse and alcoholism have been reported as causes of zinc deficiency due to increased urinary excretion, poor diet, and impaired zinc absorption [7].

Finally, extensive cutaneous burns are at a higher risk of developing zinc deficiency due to excessive skin loss and a hypercatabolic state [24,37].

#### 5.3.6. Other Causes

Down syndrome and congenital thymus defects are associated with zinc deficiency for reasons that remain unknown [2].

## 6. Differential Diagnosis

### 6.1. Other Cutaneous Diseases

Differential diagnosis should include other skin disorders that may resemble the characteristic lesions of AE and other acquired zinc deficiencies. Among these, psoriasis, severe atopic dermatitis, oral lichen planus, Behçet’s disease, autoimmune bullous diseases (pemphigus vulgaris and bullous pemphigoid), inherited bullous diseases (inherited epidermolysis bullosa), and hyperpigmentation (melasma) may present symmetrical lesions similar to those present in zinc deficiencies [4,8,9,13]. In addition, alopecia universalis may be confounded [21]. However, because zinc is deeply involved in the regulation of the immune system, it is likely that zinc deficiency may play a role in the development of these inflammatory and autoimmune disorders, and it is not uncommon for patients affected by these pathologies to present with low levels of serum zinc, with overlapping clinical findings [3,8,9,17].

Other nutritional dermatoses, such as biotin deficiency and pellagra (niacin deficiency), must be included in the differential diagnosis because they can show AE-like lesions. However, patients with pellagra present a unique phenomenon of photosensitivity, which is not seen in other diseases associated with nutritional deficiencies and can aid in diagnosis [3].

### 6.2. Inherited Metabolic Diseases

Acrodermatitis enteropathica-like skin eruptions can be observed in patients with inherited metabolic diseases. For this group of diseases, the terms acrodermatitis dysmetabolica or acrodermatitis acidemica have been proposed [1,13,38].

Among these inborn errors of metabolism, methylmalonic aciduria, propionic acidemia, maple syrup urine disease (MSUD), glutaric acyduria type I, ornithine transcarbamylase deficiency, citrullinemia, phenylketonuria, and hereditary biotin deficiency due to biotinidase deficiency have been associated with skin rashes similar to those typical of AE [3,4,23,38,39,40,41,42]. These patients may experience other severe signs and symptoms such as developmental delay, ketolactic acidosis, organic aciduria, and/or mild hyperammonemia [3,38,42].

AE-like eruptions are unrelated to zinc deficiency and are assumed to be a presentation of essential amino acid and fatty acid deficiency. Interestingly, in addition to plasma zinc dosage, alkaline phosphatase dosage should also be normal [38].

This mechanism is most likely related to a specific enzyme or metabolite deficiency, especially amino acids, which are essential for protein synthesis and metabolic decompensation [40]. Skin lesions usually resolve after normalization of amino acid levels [38,39]. With the advent of extensive metabolic screening, the management and prognosis of these diseases have significantly improved, but anecdotal cases of patients with late diagnoses and skin lesions are still described, especially in low-income countries where extensive screening is not fully available [38,39,43].

However, cases of MSUD have been described in patients with AE-like skin lesions with both specific amino acid and zinc deficiencies. Considering that patients with metabolic diseases may present poor feeding and diet restriction due to the underlying pathology, it is necessary to maintain a high index of suspicion and it is also advisable to add—to the pathology-specific enzyme/amino acid dosages—zinc, albumin, and other micronutrient dosages [13,41,44].

It has also been described that enterokinase deficiency (ED), a rare autosomal recessive disorder characterized by severe chronic diarrhea and vomiting, hypoproteinemia, edema, and failure to thrive due to defective protein absorption, may present skin lesions that overlap with those typical of AE, interestingly with normal zinc levels. Therefore, it has been proposed that ED may cause a secondary deficiency of amino acids and, therefore, skin lesions secondary to a mechanism similar to inborn errors of metabolism [45].

### 6.3. Oncological Diseases

Necrolytic migratory erythema (NME) is a cutaneous manifestation of pancreatic glucagonoma and is characterized by the resolution of skin lesions after tumor removal. Because glucagon is involved in the metabolism of amino acids, glucagon excess decreases amino acids in the serum and epidermis, leading to epidermal necrosis [3]. However, it has been hypothesized that glucagon excess and amino acid deficiency are not the only pathophysiological mechanisms, as zinc administration improves skin lesions [3].

## 7. Clinical Findings

In pediatrics, the clinical spectrum of zinc deficiency is broad, encompassing dermatological, gastrointestinal, neurological, and growth-related manifestations, given the various functions of zinc in human homeostasis [1]. Irrespective of its cause, zinc deficiency clinically presents with the classic triad of acral dermatitis, alopecia, and diarrhea; however, this pathognomonic triad is observed in less than 20% of AE cases, as reported in our case series with no evidence of diarrhea [4,6,15,46]. Table 2 shows the main clinical features of zinc deficiency.

AE may be considered the most severe form of zinc deficiency disorder, which is often fatal before the advent of zinc supplementation therapy [4].

Cutaneous findings typically include periorificial and symmetric acral dermatitis with erythematous, scaly plaques with vesiculation and crusting, alopecia, and impaired wound healing [1,6]. Patients may also present with paronychia, onychodystrophy, and onycholysis [13,14,21,23,46].

Less likely, patients may present with mucosal involvement, glossitis, cheilitis, dysgeusia, dysosmia, conjunctivitis, and blepharitis with photophobia [1,6,13,14,16,24].

The pathophysiological cause underlying skin lesions is still under debate, but it has been reported that zinc acts as a cofactor in hydrolyzing adenosine triphosphate (ATP) into adenosine monophosphate (AMP) [1,3]. Consequently, zinc deficiency in keratinocytes and Langerhans cells in the epidermis may result in local ATP excess, leading to ATP-mediated inflammation of the skin and cell apoptosis with characteristic lesions [1,3].

Zinc also exerts a protective effect against oxidative stress through the zinc finger-transactivating protein A20 and may contribute to an increased rate of re-epithelialization and cell migration for normal wound closure through the zinc-dependent matrix metalloproteinase [2].

Gastrointestinal symptoms, such as diarrhea, malabsorption, and failure to thrive, may manifest because of impaired zinc-dependent enzyme activity and immune function [1,2,15]. In children with acral symmetric dermatitis combined with malabsorption (anemia and hypoalbuminemia), cystic fibrosis should be considered [47].

Neurological findings, including developmental delay, behavioral disturbances, cognitive impairment, and other manifestations, such as hypogonadism, growth retardation, and immune system dysfunction, are less common, highlighting the critical role of zinc in neurodevelopment [15].

Untreated patients with AE may experience multiple organ failure and death [15].

A potential keystone for the differential diagnosis among different zinc deficiencies may be the timing of onset. AE is classically present in bottle-fed infants at 4–10 weeks or in breastfed infants shortly after weaning, whereas TNZD typically occurs earlier in exclusively breastfed infants, indicating insufficient zinc in breastmilk, and often resolves after weaning [1,2,4,5,6,20,23]. Skin lesions compatible with AE or TZDN, associated with zinc deficiency, and with onset in the first 4–6 months of life must, therefore, always suggest a possible underlying genetic cause. However, our case series shows that the onset can be even later. However, these clinical cases underline that whatever the cause, zinc deficiency has overlapping clinical manifestations.

TZND is probably underdiagnosed and is considered a rare disease, and few cases have been described. As the clinical presentations are often subtler than the classically described constellation of signs and symptoms, the molecular basis of these two genetic zinc deficiency syndromes is likely to be more complex than a single gene mutation [4,5,22].

In other acquired zinc deficiencies, anamnestic information is fundamental because it can suggest a previous pathology capable of determining a zinc deficiency or a dietary deficit, especially in areas of high-cereal and low-animal protein diets [2,20,33].

## 8. Laboratory Findings

The dosage of plasma or serum zinc remains the best, albeit imperfect, biomarker for zinc status [1,4,8]. Its levels serve as a cornerstone in the diagnostic workup, although interpretation may be confounded by factors such as acute or chronic illness or inflammation responding as a negative acute-phase reactant and being transported from the serum to the liver [1,2,4,7,8]. Moreover, its levels fluctuate depending on the meal and time of day [4,8].

Zinc is an essential coenzyme of metalloenzymes, including alkaline phosphatase, alcohol dehydrogenase, and other digestive enzymes [2,6,7,14]. Therefore, a low dosage of alkaline phosphatase may be a surrogate marker and an additional diagnostic clue for diagnosis, as well as an indirect index of response to therapy [1,2,13,23,24,46].

Given that zinc is bound to albumin, hypoalbuminemia may mimic zinc deficiency owing to decreased zinc-binding capacity. Therefore, in these cases, low zinc levels should be interpreted with caution and should always be correlated with the patient’s clinical presentation [1,7,8,24].

It must always be considered that the blood sample for zinc testing can be contaminated by skin traces of zinc or by tools (needles and vials), and the results may be falsely elevated if the sample is hemolyzed [7,8]. Therefore, it is advisable to carry out sampling in the morning, during fasting, using a stainless-steel needle and a vial free of trace elements [1,2,48].

Finally, it must be emphasized that the plasma zinc concentration is often normal in mild primary zinc deficiency, which can, therefore, be underdiagnosed. In addition, it is essential to remember that in cases of iron deficiency anemia or other nutritional deficiencies due to poor diet, there could likely be an associated mild-to-moderate zinc deficiency; therefore, an empirical course of zinc supplementation may be recommended [2,4].

## 9. Histological Findings

Skin biopsy in zinc deficiency dermatitis is nonspecific and usually indistinguishable from other types of deficiency dermatitis, such as pellagra or cutaneous manifestations of various metabolic inherited diseases [7,27,28,36,41]. Moreover, histopathology may vary according to the progression of the lesion [2,11,28].

Zinc deficiency dermatosis is initially characterized by alternating orthokeratosis and parakeratosis with a thinner granular layer, acanthosis, and focal acantholysis [2,7]. As confirmed in Case 2, dermal capillaries may be dilated with a sparse dermal lymphohistiocytic infiltrate. Later stages are marked by ballooning degeneration of keratinocytes with pale cytoplasm, suggesting necrolysis [2,11,13]. Chronic lesions sometimes exhibit a psoriasiform pattern [1,2,11,13]. Thus, skin biopsies are primarily used to rule out other disorders [1].

## 10. Management

Zinc supplementation is the cornerstone of therapy for acquired zinc deficiency and is administered orally or parenterally, depending on the severity and underlying cause [1,2,3].

For AE, TSZD, and other secondary zinc deficiencies, therapy consists of zinc supplementation, which leads to clinical and dermatological improvement within days, even before a significant increase in serum zinc levels [1,2,3]. However, the treatment and dosage of zinc supplementation are determined by the cause of the disorder [1,2].

The difference between AE, TSZD, and other zinc deficiencies is crucial for defining zinc treatment duration. In TSZD a 0.5–1 mg/kg/die supplementation may be sufficient, and it can be stopped shortly after 3–4 months or after alimentary diversification [1,6,21,49]. In contrast, in AE, a 1 mg/kg/day dose may not be sufficient, requiring higher doses and lifelong zinc replacement [1,2,4,17,49].

Patients with hereditary AE should take elemental zinc at a dose of 3 mg/kg per day, and plasma zinc levels should be measured every 3–6 months [2,4]. The dosage must be adjusted according to zinc plasma levels to avoid dosages remaining constant over time, leading to relapse [48].

Moreover, relapse after discontinuation of zinc therapy may suggest an inherited deficiency and should be considered in adolescent or adult patients with relapsed typical lesions who have discontinued zinc supplementation [6,24,31,46,50].

Because of possible interactions, copper, iron, vitamin A, and vitamin D levels should also be monitored and co-administered if reduced [2,4,8,16,24].

However, recently, new therapeutic options have been proposed, with no apparent effect on either copper or iron serum levels and no need for daily supplementation, which is the main reason for long-term suspension [51].

In the management of a patient with a zinc deficiency-related disease, whether inherited or acquired, the risk of bacterial or fungal superinfection of skin lesions must always be considered [1]. These infections are likely due to loss of the skin barrier, impaired lymphocyte generation, and zinc-mediated function [1,3]. The main pathogens are *methicillin-resistant Staphylococcus Aureus*, *Klebsiella Pneumoniae*, *Candida* spp., *and Pseudomonas Aeruginosa*, which may lead to invasive infections and sepsis. It is not uncommon for the patient to present to the PED for febrile infection rather than for skin lesions, as shown in Case 1 [5,7,10,11,13,14,18,22,28,42,47].

Thus, although AE is treatable, early recognition and prompt supplementation therapy are mandatory and may prevent systemic consequences encompassing neurological development and immune regulation [16]. Furthermore, it has been reported that zinc supplementation may result in a slight reduction in mortality and the incidence of all-causes diarrhea due to respiratory tract infections and malaria [12]. Thus, physicians should maintain high clinical suspicion and consider measuring zincemia, even in the absence of a complete triad of typical AE symptoms or in the presence of other confounding features, such as fever [6].

Zinc is generally considered non-toxic, even at high dosages [4,8]. However, acute zinc overdose may be associated with undesirable adverse effects, including diarrhea, nausea, vomiting, lethargy, mild headaches, and fatigue [1,2,8,13]. Chronic overdose may lead to neutropenia, leukopenia, copper and/or iron deficiency, anemia, growth retardation, decreased high-density lipoprotein levels, and increased low-density lipoprotein levels [2].

## 11. Conclusions

Zinc deficiency can be a diagnostically challenging condition. Timely recognition and targeted intervention are imperative for optimizing clinical outcomes, mitigating long-term complications, and improving patient quality of life.

Although zinc deficiency and its consequences are linked to poverty, it is not possible to know the effective incidence, as the data for some remote, rural areas are not known. Future efforts must be made to enable all children in every country to receive preventive zinc supplementation.

Physicians should consider measuring serum zinc levels in any infants with irritability, chronic diarrhea, alopecia, and typical rashes in the periorificial, perineal, and acral distribution, even in the presence of confounding symptoms, such as fever.

Whatever the cause, the lack of zinc supplementation may lead to an increased risk of morbidity and mortality in young children. Thus, the prompt identification of zinc deficiency should be considered as a medical emergency.

## Figures and Tables

**Table 1:** Etiology of zinc deficiency in children [1,2].

**Inadequate intake**	Low maternal zinc diet in breastfed infants Transient neonatal zinc deficiency Parenteral nutrition Anorexia nervosa Bulimia Malnutrition *Kwashiorkor* *Alcoholism* Veganism, vegetarianism, ketogenic diet Food faddism
**Increased losses**	Acute and recalcitrant diarrhea Post-surgical intestinal fistulas * Urine losses *Nephrotic syndrome* *Glycosuria and diabetes mellitus* *Diuretics* Burns Excessive sweating Hemodialysis
**Malabsorption**	Acrodermatitis enteropathica Cystic fibrosis Celiac disease Inflammatory bowel disease Short bowel syndrome * Liver and/or pancreatic insufficiency Bariatric surgery * Iatrogenic causes *Penicillamin* *High intake of phytate* *Copper supplementation or deficiency* *Iron supplementation or deficiency* *Valproate*
**Increased requirement**	Pregnancy Breastfeeding Preterm babies/IUGR/SGA Hypercatabolism
**Unclear mechanism**	Down syndrome Congenital thymus defect

* May cause zinc deficiency, malabsorption, and increased losses; IUGR = intrauterine growth retardation; SGA = small for gestational age.

**Table 2:** Clinical features of zinc deficiency [2,8].

**Mucosal and cutaneous features**	Delayed wound healing Alopecia and telogen effluvium Acral skin lesions (erythema, erosions, ulcerations, bullae) Stomatitis Paronychia Onycodystrophia Blepharitis Cheilitis and glossitis
**Immune system features**	Impaired cell-mediated immune function Recurring infection
**Gastrointestinal features**	Diarrhea Anorexia Hypogeusia
**Endocrine features**	Growth retardation Delayed puberty Hypogonadism
**Central nervous system features**	Neurosensory development retardation Behavioral disorders Intentional tremor Impaired concentration Night blindness Anosmia Dysarthria
**Musculoskeletal features**	Decreased lean body mass Increased risk of bone fractures
**Pregnant state**	Delayed fetal growth Low birth weight Preterm labor

## Data Availability

No new data were created.
